# Sphk1 promotes breast epithelial cell proliferation via NF-κB-p65-mediated cyclin D1 expression

**DOI:** 10.18632/oncotarget.13013

**Published:** 2016-11-02

**Authors:** Shifei Li, Yan Zhou, Xiaodong Zheng, Xiujuan Wu, Yueyang Liang, Shushu Wang, Yi Zhang

**Affiliations:** ^1^ Breast Disease Center, Southwest Hospital, Third Military Medical University, Chongqing 400038, China

**Keywords:** Sphk1, NF-κBp65, CyclinD1, cell proliferation

## Abstract

Lipid metabolism is crucially involved with the promotion of malignant progression and metastasis in various cancers. Growing evidence suggests that many types of cancers express high levels of sphingosine kinase 1 (Sphk1), which is known to mediate cell proliferation We hypothesized that Sphk1/sphingosine-1-phosphate (S1P) signaling contributes to tumor progression. In MCF10A and MCF10A-Sphk1 breast epithelial cells, we used TNF-α to activate the Sphk1/S1P pathway and the measured expression levels of NF-κBp65 and cyclin D1 mRNA and protein in the presence and absence of an NF-κB-p65 inhibitor. Chromatin immunoprecipitation assays were performed to determine whether NF-κB-p65 binds to the cyclin D1 promoter. We found that overexpression of Sphk1 induced NF-κB-p65 activation, increased expression of cyclin D1, shortened the cell division cycle, and thus promoted proliferation of breast epithelial cells. These findings provide insight into the mechanism by which an Sphk1/NF-κB-p65/cyclin D1 signaling pathway mediates cell proliferation.

## INTRODUCTION

Sphingosine kinase 1 (Sphk1), a key enzyme involved in membrane lipid metabolism, is highly expressed in breast cancer [1]. Moreover, Sphk1 overexpression correlates with a poor clinical prognosis in breast and other cancers [2]. Sphingosine-1-phosphate (S1P), the synthesis of which is catalyzed by Sphk1, functions as a second messenger mediates increases cell proliferation, apoptosis, differentiation and tumorigenesis [3–5]. For example, Sphk1 activation promotes the proliferation of intestinal adenoma cells, while suppression of Sphk1 expression inhibits cell proliferation [6]. In addition, Sphk1 expression is significantly higher in cervical cancer than in normal tissues, while Sphk1 inhibitors reduce cancer cell survival and promote apoptosis among cancer cells. Most importantly, the overall survival rate among cervical cancer patients expressing low levels of Sphk1 is much better than among those expressing high levels of the enzyme [7].

Coexpression of Sphk1 and cyclinD1 has been detected in breast tissue, in abnormally growing cells and in breast cancer cells. Cyclin D1 overexpression induces cell growth and transformation and tumorigenesis by shortening cell cycle G1 phase and thus promoting entry into S phase [8, 9]. However, overexpression of cyclin D1 requires the binding of an activated transcription factor to its promoter to enhance its transcription activation. Previous studies have shown that activation of Sphk1 signaling leads in turn to activation or inhibition of various transcription factors, including NF-κB, E2F, c-Myc and Sp1, which then enhance or suppress cell proliferation, apoptosis and/or inflammation [10–12]. Although the transcription factor via which Sphk1 signaling increases cyclinD1 expression has not yet been identified, that levels of the p65 subunit of NF-κB are increased in breast cancer specimens, and its expression is associated with cell progression [13]. In the present study, the Sphk1/S1P signaling pathway was activated to enhance NF-κB-p65 and cyclin D1 expression, which in turn promoted breast epithelial cell proliferation.

## RESULTS

### Sphk1 overexpression enhances proliferation of MCF10A cells

**Figure 1:**
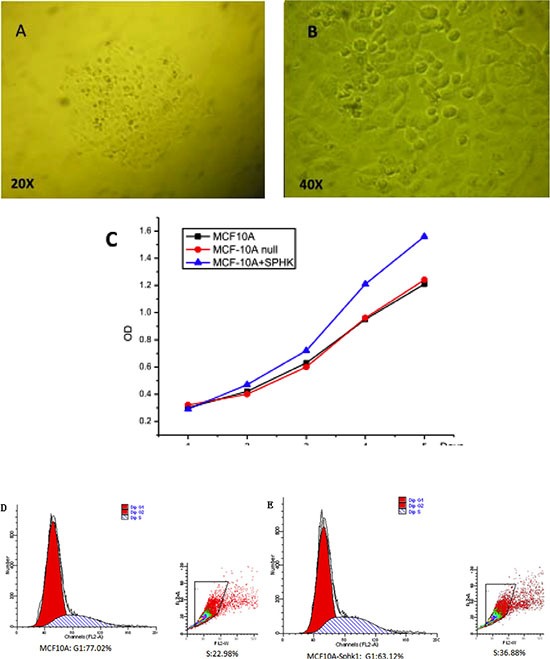
**(A and B)** Isolated single MCF10A clones after transduction with a lentivirus vector. Single clones were visualized through microscopy. (**C**) Sphk1 overexpression promoted the proliferation of MCF10A breast epithelial cells. (**D** and **E**) Effect of Sphk1 overexpression on the cell cycle.

Sphk1 converts sphingosine to S1P, which promotes cell growth, proliferation and survival, and is a key promoter in cancer. Sphk1 was stably overexpressed in MCF10A-derived MCF10A-Sphk1 cells (Figure 1A and 1B). Through cell counting (Table 1), we found that MCF10A-Sphk1 cells proliferated more rapidly than the parental MCF10A cells (Figure 1C). Moreover, it appeared the rapid proliferation reflected a shortened G1 phase in the cells overexpressing Sphk1 (Figure 1D and 1E).

**Table 1:** The ability of cell proliferation

Sample/OD	24 h	48 h	72 h	96 h	120 h
MCF10A	0.337662	0.41836	0.603873	0.938784	1.225147
	0.338428	0.428714	0.610921	0.928827	1.224144
	0.339872	0.41985	0.609754	0.930389	1.222812
MCF10A null	0.335193	0.408657	0.570812	0.931589	1.254508
	0.336465	0.407132	0.57104	0.931268	1.265238
	0.33612	0.408977	0.58234	0.931522	1.252223
MCF10A-Sphk1	0.315432	0.471853	0.734865	1.206599	1.574735
	0.304059	0.4716327	0.730413	1.208623	1.581125
	0.312345	0.473487	0.731081	1.21617	1.580085

### Sphk1/S1P pathway activation promotes expression of NF-κB-p65 and cyclin D1

**Figure 2:**
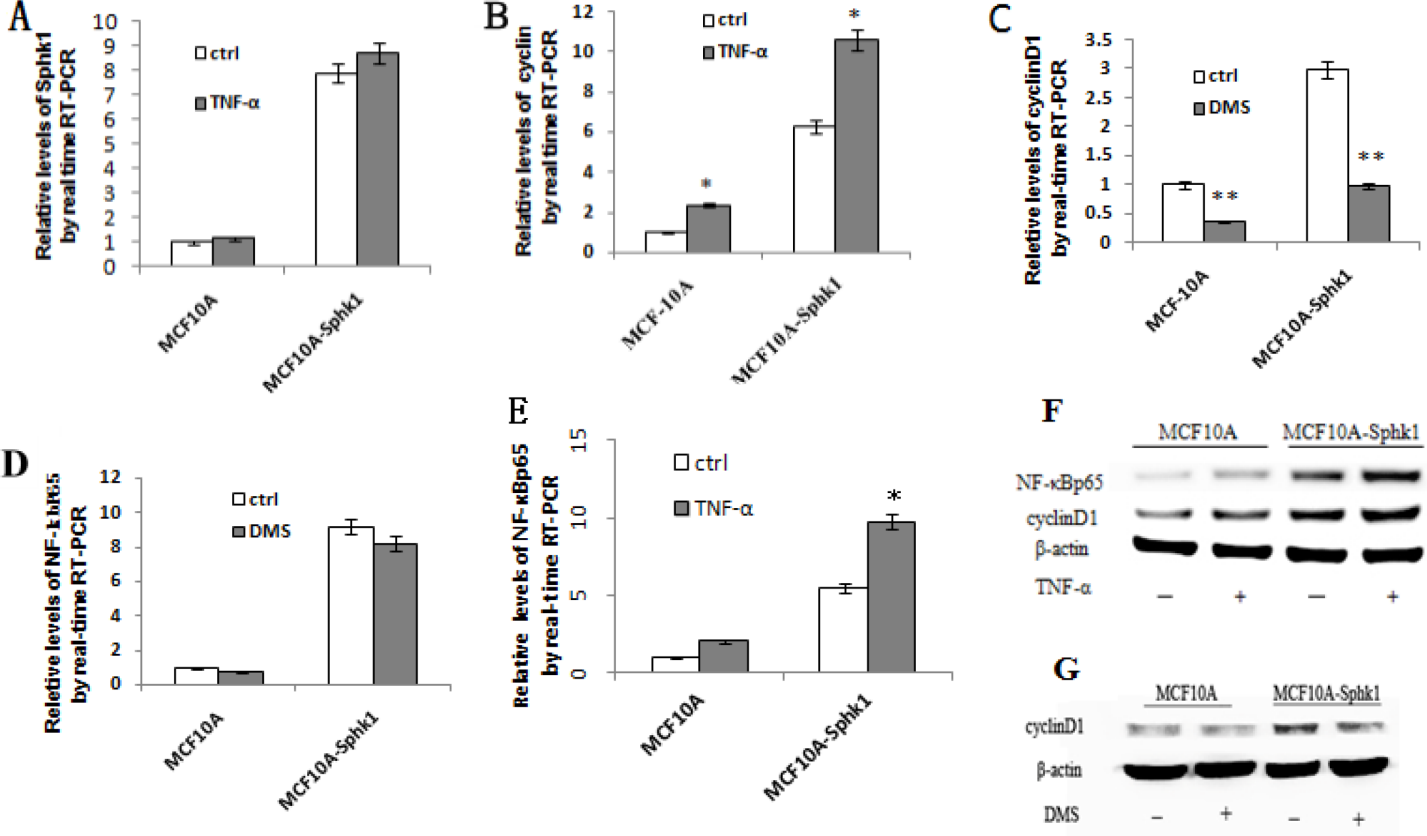
Activation of Sphk1/S1P signaling upregulated expression of cyclin D1 and NF-κB-p65 The effects were blocked by Sphk1 inhibition. Sphk1/S1P signaling was activated in MCF10A and MCF10A-Sphk1 cells using TNF-α, after which cells were collected for RT-PCR (**A**, **B**, **E**) and Western blotting (**F**) to quantify the expression of cyclin D1 and NF-κB-p65. The cells were also stimulated in the presence of the Sphk1 inhibitor DMS and then collected for RT-PCR (**C** and **D**) and Western blotting (**G**). *p < 0.05, **p < 0.01.

**Figure 3:**
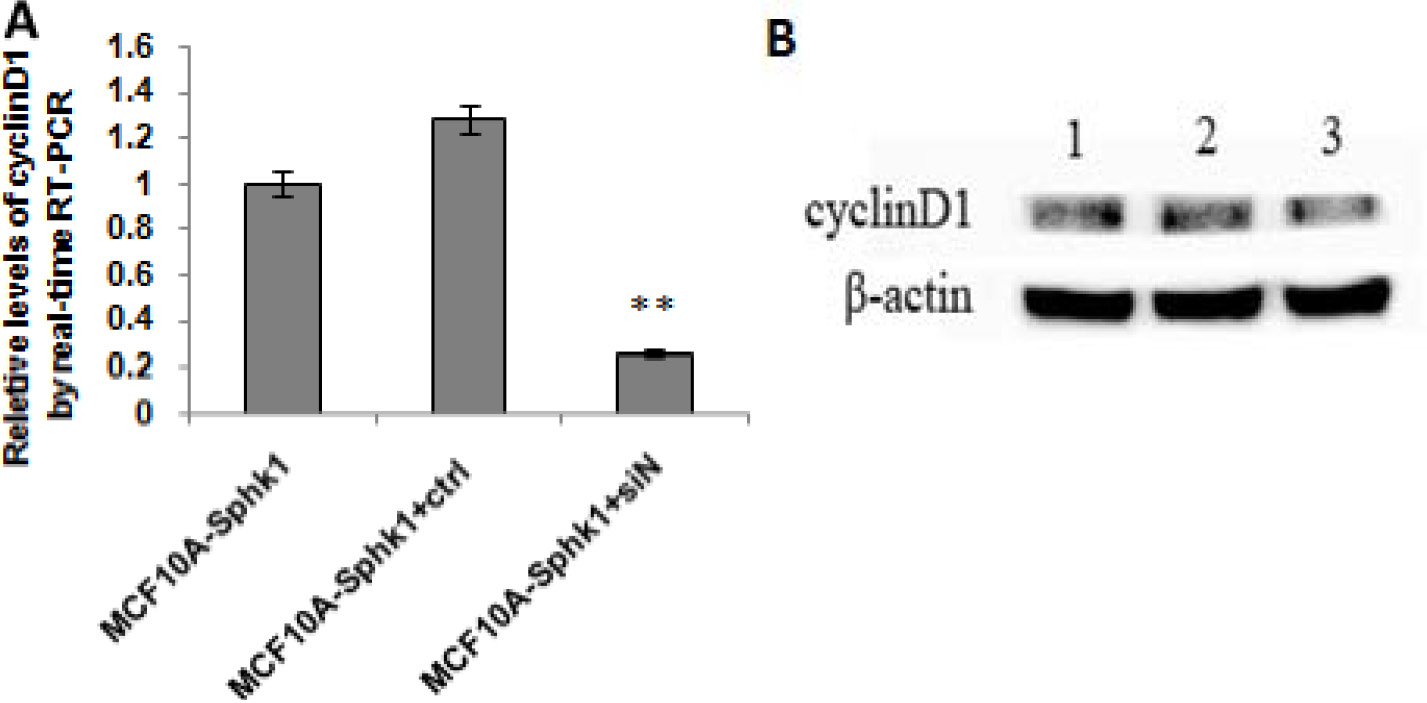
Activated Sphk1/S1P signaling and inhibited the expression of NF-κBp65 MCF10A and MCF10A-Sphk1 cells were treated with TNF-α for 12 h and then transfected with NF-κB-p65 siRNA for 24 h. They were then collected for RT-PCR (**A**) and Western blotting (**B**) to quantify the expression of cyclin D1. Lane 1, MCF10A-Sphk1; lane 2, MCF10A-Sphk1+control siRNA; lane 3, MCF10A-Sphk1+TNF-a + NF-κB-p65 siRNA. **p < 0.01.

In both MCF10A and MCF10A-Sphk1 cells, Sphk1/S1P signaling was activated by TNF-α. Western blot and RT-PCR analyses showed that expression of Sphk1, cyclin D1 and NF-κB-p65 was significantly higher in MCF10A-Sphk1 than MCF10A cells (Figure 2A, 2B, 2E and 2F). This suggests TNF-α activated the Sphk1/S1P pathway, thereby enhancing NF-κB-p65 activation and promoting expression of cyclin D1 and shortening cell cycling. Consistent with that idea, when cells were treated with the Sphk1 inhibitor DMS, the reductions in the levels of cyclin D1 and NF-κB-p65 mRNA and protein were significantly greater in MCF10A-Sphk1 than MCF10A cells (Figure 2C, 2D and 2G). Similarly, when we using siRNA to suppress expression of NF-κB-p65 in MCF10A and MCF10A-Sphk1 cells, the reductions in the levels of cyclin D1 mRNA and protein were greater than those in MCF10A cells (Figure 3). These results indicate that the co-expression of NF-κB-p65 and cyclinD1 correlated with activation of Sphk1/S1P signaling, and that Sphk1/S1P signaling increases cyclin D1 expression via the transcription factor NF-κB.

### Sphk1/S1P signaling promotes NF-κB-p65 binding to the cyclin D1 promoter and increases gene transcription

**Figure 4:**
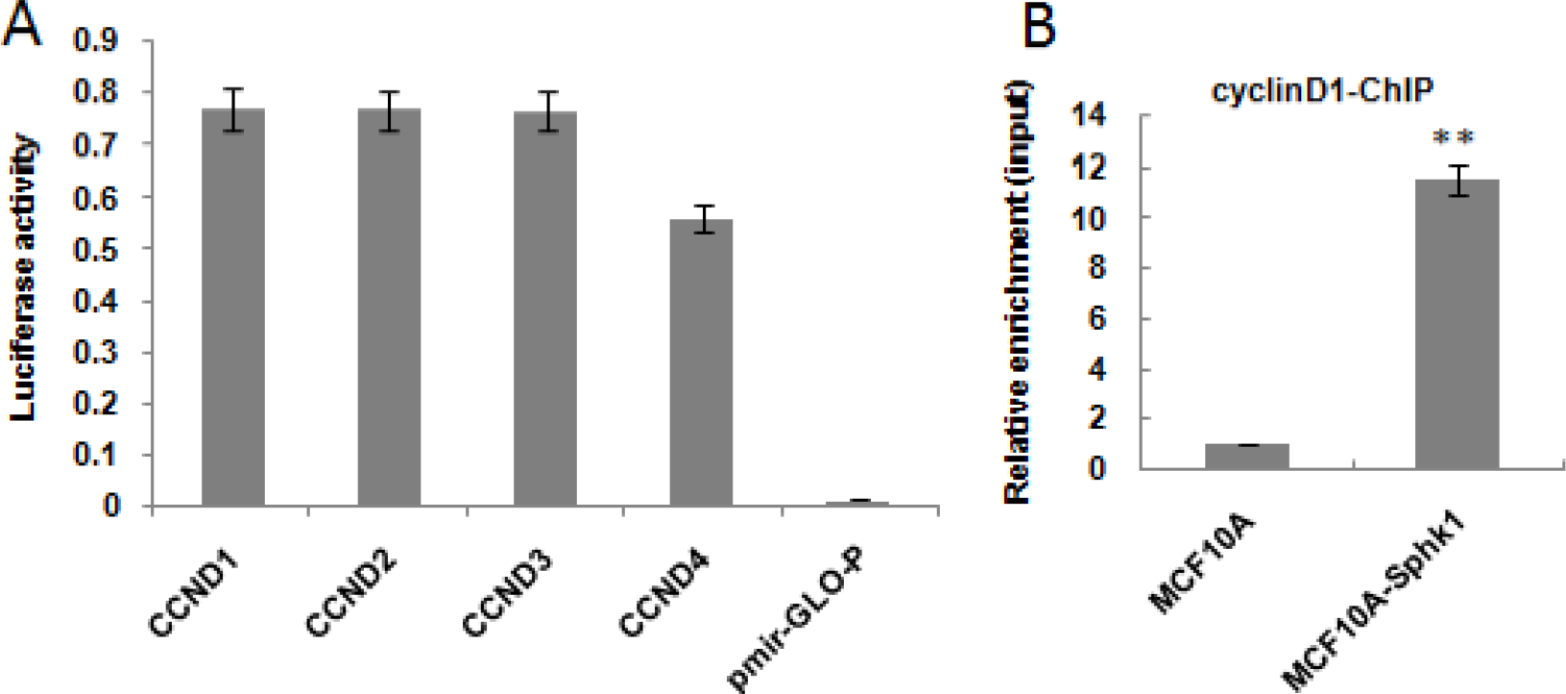
Activated Sphk1/S1P promotes NF-kB-p65 binding to the cyclin D1 promoter The effect of deleting the cyclin D1 promoter was assessed using a luciferase reporter system (**A**). ChIP-seq was performed to determine whether NF-κB-p65 bound to the cyclin D1 promoter (**B**). **p < 0.01

Online prediction analysis software identified 8 potential NF-κB bindings sites in the cyclin D1 promoter. We therefore conducted ChIP assays to determine whether NF-κB-p65 binds to the CyclinD1 promoter. The ChIP assays confirmed that NF-κB-p65 bound to the cyclin D1 promoter to increase transcription and gene expression. Then to better identify the NF-κB-p65 binding site, we generated multiple truncation mutants (CCND1-4) and used a luciferase reporter system to detect the activation of mutants. The results showed that the activities of CCND1-3, truncated at positions −1800, −1500 and −900, respectively, were equal to or higher than wild-type cyclin D1. By contrast, the activity of CCND4, truncated at position −600, was significantly weaker than the others (Figure 4A).

## DISCUSSION

Excessive cell proliferation contributes to malignant cell transformation. Earlier studies showed that activated NF-κB binds to the cyclin D1 promoter, stimulating its expression [15], and that NF-κB controls miR-21-induced transcription of cyclin D1 in renal cancer cells [16]. In addition, Sphk1/S1P signaling is reportedly involved in cell proliferation, survival and cytoskeletal rearrangement [17, 18]. S1P is generated through Sphk-catalyzed phosphorylation of sphingosine, and the Sphk1/S1P pathway has been implicated in tumor progression [19–21]. Sphk1 expression is normally low in breast epithelial tissues, and its overexpression in breast epithelial cells significantly enhances the cells' proliferation (Figure 1B).

Cell proliferation is governed in part by the cyclins, a family cell cycle proteins. Cyclin D belongs to a subfamily that increases cell cycling by binding to cyclin-dependent kinase (CDK)-4 [22]. For example, cyclin D1, which enhances transcriptional regulation in several human cancers [23], promotes progression through the G1-S phase of the cell cycle by binding to CDK-4 to phosphorylate and inactivate retinoblastoma protein and release E2F transcription factors [24]. Thus, overexpression of cyclin D1 promotes cell proliferation. In the present study, we observed that in MCF10A-Sphk1 cells, levels of cyclin D1 expression were higher than in MCF10A cell (Figure 2B and 2F). Furthermore, the increase in cyclin D1 led to increased cell proliferation.

Activated NF-κB-p65 translocates from cytoplasm to nucleus and targets DNA sequences to modulate gene transcription. Moreover, studies suggest NF-κB-mediated cyclin D expression contributes to the progression of both glioma [25] and renal cancer cell [26]. Consistent with those findings, we observed that activation of Sphk1 signaling increases expression of NF-κB-p65 and, in turn, cyclin D1, leading to enhanced proliferation of MCF10A-Sphk1 breast epithelial cells. This suggests that in the malignant transformation of breast epithelial cells, activation of Sphk1/S1P signaling enhances NF-κB-p65 expression and activation. The activated NF-κB-p65 presumably relocates to the nucleus, binds to the cyclin D1 promoter to increase it expression and promote transition from G1 to S phase, which would increase cell proliferation. Collectively then, our findings indicate that a Sphk1/S1P/NF-κB-p65/cyclin D1 signaling pathway plays a key role in the proliferation and malignant transformation in breast epithelial cells.

## MATERIALS AND METHODS

### Cell transfection

The MCF10A human breast epithelial cell line was obtained from the cell bank of the Chinese Academy of Science, Shanghai, China. To establish the stable MCF10A-Sphk1 cell line, the cells were cultured in mammary epithelial cell growth medium (MEGM) (Clonetics Corp, US) supplemented with 100 ng/ml cholera toxin. They were then plated in 6-well plates at a density of 3 × 10^5^cells/well in 2 ml of fresh culture medium and incubated for 24 h. Thereafter, 500 μl of LV-TOPO-Sphk1(we purchased the vector and packaged the lentivirus on its own) in 1 ml of fresh culture medium were added, and the cell cultures were incubated for an additional 24 h or 48 h. Cells stably expressing Sphk1 were then selected for 4 to 6 days in medium containing blasticidin (5 mg/ml).

### Cell culture and TNF-α treatment

MCF10A and MCF10A-Sphk1 cells were maintained in MEGM containing 100 ng/ml cholera toxin at 37°C and under 5% CO_2_ in air. The cells were then plated in 6-well plates at a density of 3 × 10^5^ cells/well in fresh culture medium and incubated for 12 h, after which TNF-a (50 ng/ml) was added for 24 h. The cells were collected for Western blotting and reverse transcription-polymerase chain reaction (RT-PCR).

### Western blotting

For a total protein analysis, treated and untreated cells were harvested and lysed in T-PER Tissue Protein Extraction Reagent (Pierce Chemical Company) supplemented with phenylmethylsulfonyl fluoride and incubated at 4°C for 30 min. The lysates were then centrifuged at 12,000 rpm for 20 min at 4°C, the supernatant was collected, and equal amounts of protein were separated on SDS-PAGE (10% gel) and electrotransferred onto PVDF membranes (Millipore). After blocking with 5% skimmed milk in TBST, the membranes were incubated with a primary antibody and then stained with the appropriate horseradish peroxidase-conjugated secondary antibody. Immunoreactive proteins were detected using SuperSignal West Dura Extended Duration Substrate (Thermo Scientific), after which the reactive bands were detected using enhanced chemiluminescence reagents (Thermo). To ensure the equal loading of samples in each lane, membranes were stripped and re-probed with an anti-β-actin antibody, as previously described [14].

### RNA extraction and quantitative real-time PCR analysis (RT-qPCR)

Total RNA was extracted using RNAiso reagent (Takara) according to the manufacturer's instructions and quantified using a NanoDrop spectrophotometer (Thermo Scientific). For cyclinD1 and NF-κB-p65 detection, 300 ng of total RNA was converted to cDNA using specific primers, and the cDNA was amplified using SYBR Premix Ex TaqTM (TaKaRa). The PCR protocol entailed 3 min at 95°C, 40 cycles of 20 s at 95°C, 30 s at 60°C and 20 s at 72°C, followed by 7 min at 72°C. The expression levels of cyclin D1 and NF-κB-p65 were normalized to those of β-actin. The relative mRNA levels were calculated using the comparative threshold cycle method [14].

### siRNA transfection

To knock down cyclin D1 or NF-κBp65 with cyclin D1 siRNA or NF-κBp65 siRNA, chemosynthesized siRNAs (SANTA) were dissolved in 150 ml of RNase-free water to a reserve concentration of 20 μM. MCF10A-Sphk1 cells were then seeded into 6-well plates at a density of 1.2 × 10^5^cells/well and incubated for 24 h, after which the cells were transfected siRNA or negative control at a final concentration of 50 nM. Cells were allowed to grow for 48 h after transfection and were then treated as indicated.

### Chromatin immunoprecipitation (ChIP) assays

ChIP was performed using a commercially available Chromatin Immunoprecipitation Kit (Upstate Biotechnology) according to the manufacturer's instructions. Briefly, cells (2.5 × 10^6^/immunoprecipitation) were treated with 1% (w/v) formaldehyde for 10 min at room temperature, and cross-linking was halted with 0.125 M glycine on ice. The cross-linked chromatin was then sonicated and immunoprecipitated using rabbit polyclonal anti-NF-κB-p65 antibody. After the revering the cross-linking, the precipitated DNA was analyzed by PCR (35 cycles) with one primer pair and one negative primer pairs for regions lacking NF-κB-p65 binding sites.

### Cell culture and N, N-dimethylsphingosine (DMS) processing

MCF10A and MCF10A-Sphk1 cells were incubated in MEGM supplemented with 100 ng/ml cholera virus at 37°C under 5% CO_2_/Air. The cells were then harvested, seeded into 6-well plates at 3 × 10^5^ cells/well and incubated for 12 h, after which DMS (0.5 μM) was added, and the cells were incubated for an additional 24 h in Western blotting and RT-PCR analyses.

### Vectors

The promoter sequence for cyclin D1 (GeneBank accession No. NM_007631) was identified, and potential binding sites for NF-κB-p65 were found to synthesize primers (cyclin D1 F:ACCATTCCCTTGACTGCCGA R:GGAGGGTGGGTTGGAAATGA). The promoter region was then cloned, purified and migrated into Pmir-GLO-P (TOYOBO) vector using T4 Ligase (TOYOBO). *Escherichia coli* DH10B was then transformed with the vector, and sequencing was used to identify correct single clones.

### Mutation of cyclinD1 promoter

A 500-bp deletion in the cyclin D1 promoter was generated at −1800, −1500, −900 and −600 using the following primer sequence information: CCND1-F, 5′CCCTTACCAGTTTCTTCGGG3′; CCND 2-F, 5′GCTAGGTTACTGCTGTAAGC3′; CCND3-F, 5′GG CCTTAGCTTATGGCCCCATTA3′; CCND4-F, 5′GGAT CCGGGCTCACATGA3′; CCND-R, 5′TTCCATGGC GCGGCCGCCTGGGGA3′.

The respective truncated promoters were separately ligated into Pmir-GLO-P vector to produce CCND1-4 plasmids used to transfect MCF10A and MCF10A-Sphk1 cells. The promoter activity was assessed using a luciferase reporter system (Promega).

## References

[1] Zhang Y, Zhou Y, Wang Z, Chen Q, Jiang J (2010). Expression of sphingosine kinase 1 in atypical hyperplasia of the breast and breast cancer and its significance. Chinese Journal of Breast Disease (Electronic Version).

[2] Li J, Song Z, Wang Y, Yin Y, Liu Y, Yuan R, Nan X (2016). Overexpression of SphK1 enhances cell proliferation and invasion in triple-negative breast cancer via the PI3K/AKT signaling pathway. Tumour Biol.

[3] Pappu R, Schwab SR, Cornelissen I, Pereira JP, Regard JB, Xu Y, Camerer E, Zheng YW, Huang Y, Cyster JG, Coughlin SR (2007). Promotion of lymphocyte egress into blood and lymph by distinct sources of sphingosine-1-phosphate. Science.

[4] Okada T, Kajimoto T, Jahangeer S, Nakamura S (2009). Sphingosine kinase/sphingosine 1-phosphate signalling in central nervous system. Cell Signal.

[5] Kohno M, Momoi M, Oo ML, Paik JH, Lee YM, Venkataraman K, Ai Y, Ristimaki AP, Fyrst H, Sano H, Rosenberg D, Saba JD, Proia RL, Hla T (2006). Intracellular role for sphingosine kinase 1 in intestinal adenoma cell proliferation. Mol Cell Biol.

[6] Wang F, He W, Fanghui P, Wang L, Fan Q (2013). NF-κBP65 promotes invasion and metastasis of oesophageal squamous cell cancer by regulating matrix metalloproteinase-9 and epithelial-to-mesenchymal transition. Cell Biol Int.

[7] Kim HS, Yoon G, Ryu JY, Cho YJ, Choi JJ, Lee YY, Kim TJ, Choi CH, Song SY, Kim BG, Bae DS, Lee JW (2015). Sphingosine kinase 1 is a reliable prognostic factor and a novel therapeutic target for uterine cervical cancer. Oncotarget.

[8] Zhong Z, Yeow WS, Zou C, Wassell R, Wang C, Pestell RG, Quong JN, Quong AA (2010). Cyclin D1/cyclin-dependent kinase 4 interacts with filamin A and affects the migration and invasion potential of breast cancer cells. Cancer Res.

[9] Nava VE, Hobson JP, Murthy S, Milstien S, Spiegel S (2002). Sphingosine kinase type 1 promotes estrogen-dependent tumorigenesis of breast cancer MCF-7 cells. Exp Cell Res.

[10] Hazar-Rethinam M, de Long LM, Gannon OM, Topkas E, Boros S, Vargas AC, Dzienis M, Mukhopadhyay P, Simpson F, Endo-Munoz L, Saunders NA (2015). A novel E2F/sphingosine kinase 1 axis regulates anthracycline response in squamous cell carcinoma. Clin Cancer Res.

[11] Jiang P, Smith AD, Li R, Rao JN, Liu L, Donahue JM, Wang JY, Turner DJ (2013). Sphingosine kinase 1 overexpression stimulates intestinal epithelial cell proliferation through increased c-Myc translation. Am J Physiol Cell Physiol.

[12] Zhu L, Wang Z, Lin Y, Chen Z, Liu H, Chen Y, Wang N, Song X (2015). Sphingosine kinase 1 enhances the invasion and migration of non-small cell lung cancer cells via the AKT pathway. Oncol Rep.

[13] Liu S, Tan WY, Chen QR, Chen XP, Fu K, Zhao YY, Chen ZW (2008). Daintain/AIF-1 promotes breast cancer proliferation via activation of the NF-kappaB/cyclin D1 pathway and facilitates tumor growth. Cancer Sci.

[14] Li J, Shan F, Xiong G, Chen X, Guan X, Wang JM, Wang WL, Xu X, Bai Y (2014). EGF-induced C/EBP participates in EMT by decreasing the expression of miR-203 in esophageal squamous cell carcinoma cells. J Cell Sci.

[15] Denis C, Guttridge C, Albanese JY, Reuther RG, Pestell AS, Baldwin NF-Kb controls cell growth and differentiation through transcription regulation of cycinD1. Mol Cell Biol.

[16] Amit Bera, Nandini Ghosh-Choudhury, Nirmalya Dey, Falguni Das, Balakuntalam S, Kasinath, Hana E, Abboud, Ghosh Choudhury Goutam (2013). NFKB-mediated cyclinD1 expression by microRNA-21 influences renal cancer cell proliferation. Cellular Signalling.

[17] Takabe K, Paugh SW, Milstien S, Spiegel S (2008). “Inside-Out” Signaling of Sphingosine-1-Phosphate: Therapeutic Targets. Pharmacol Rev.

[18] Zheng XD, Zhang Y, Qi XW, Wang MH, Sun P, Zhang Y, Jiang J (2014). Role of Sphk1 in the malignant transformation of breast epithelial cells and breast cancer progression. Indian J Cancer.

[19] Pyne NJ, Pyne S (2010). Sphingosine 1-phosphate and cancer. Nat Rev Cancer.

[20] Fyrst H, Saba JD (2010). An update on sphingosine-1-phosphate and other sphingolipid mediators. Nat Chem Biol.

[21] Nagahashi M, Ramachandran S, Kim EY, Allegood JC, Rashid OM, Yamada A, Zhao R, Milstien S, Zhou H, Spiegel S, Takabe K (2012). Sphingosine-1-phosphate produced by sphingosine kinase 1 promotes breast cancer progression by stimulating angiogenesis and lymphangiogenesis. Cancer Res.

[22] Bramanti V, Campisi A, Tomassoni D, Li VG, Caccamo D, Cannavo G, Curro M, Raciti G, Napoli M, Ientile R, Vanella A, Amenta F, Avola R (2008). Effect of acetylcholine precursors on proliferation and differentiation of astroglial cells in primary cultures. Neurochem Res.

[23] Bramanti V, Tomassoni D, Bronzi D, Grasso S, Curro M, Avitabile M, Li VG, Renis M, Ientile R, Amenta F (2010). Alpha-lipoic acid modulates GFAP, vimentin, nestin, cyclin D1 and MAP-kinase expression in astroglial cell cultures. Neurochem Res.

[24] Swanton C (2004). Cell-cycle targeted therapies. Lancet Oncol.

[25] Wu M, Huang C, Li X, Li X, Gan K, Chen Q, Tang Y, Tang K, Shen S, Li G (2008). LRRC4 inhibits glioblastoma cell proliferation, migration, and angiogenesis by downregulating pleiotropic cytokine expression and responses. J Cell Physiol.

[26] Bera A, Ghosh-Choudhury N, Dey N, Das F, Kasinath BS, Abboud HE, Choudhury GG (2013). NFκB-mediated cyclin D1 expression by microRNA-21 influences renal cancer cell proliferation. Cell Signal.

